# Human Papillomavirus 16-Transgenic Mice as a Model to Study Cancer-Associated Cachexia

**DOI:** 10.3390/ijms21145020

**Published:** 2020-07-16

**Authors:** Sara Peixoto da Silva, Joana M. O. Santos, Verónica F. Mestre, Beatriz Medeiros-Fonseca, Paula A. Oliveira, Margarida M. S. M. Bastos, Rui M. Gil da Costa, Rui Medeiros

**Affiliations:** 1 Molecular Oncology and Viral Pathology Group, IPO Porto Research Center (CI-IPOP), Portuguese Oncology Institute of Porto (IPO Porto), 4200-072 Porto, Portugal; peixotodasilva.sara@gmail.com (S.P.d.S.); joana.oliveira.santos@ipoporto.min-saude.pt (J.M.O.S.); rmcosta@fe.up.pt (R.M.G.d.C.); 2 Faculty of Medicine of the University of Porto (FMUP), 4200-319 Porto, Portugal; 3 Department of Veterinary Sciences, University of Trás-os-Montes and Alto Douro (UTAD), 5001-801 Vila Real, Portugal; vero.mestre11@gmail.com (V.F.M.); fonsecabeatriz@live.com.pt (B.M.-F.); pamo@utad.pt (P.A.O.); 4 Center for the Research and Technology of Agro-Environmental and Biological Sciences (CITAB), University of Trás-os-Montes and Alto Douro (UTAD), 5001-911 Vila Real, Portugal; 5 LEPABE—Laboratory for Process Engineering, Environment, Biotechnology and Energy, Faculty of Engineering, University of Porto, 4200-465 Porto, Portugal; mbastos@fe.up.pt; 6 Postgraduate Programme in Adult Health (PPGSAD), Tumour and DNA Biobank, Federal University of Maranhão (UFMA), 65080-805 São Luís, Brazil; 7 Virology Service, Portuguese Oncology Institute of Porto (IPO Porto), 4200-072 Porto, Portugal; 8 CEBIMED, Faculty of Health Sciences of the Fernando Pessoa University, 4249-004 Porto, Portugal; 9 Research Department of the Portuguese League Against Cancer—Regional Nucleus of the North (Liga Portuguesa Contra o Cancro—Núcleo Regional do Norte), 4200-177 Porto, Portugal

**Keywords:** cancer cachexia, HPV16, K14-HPV16, mouse model, DMBA, wasting syndrome

## Abstract

Cancer cachexia is a multifactorial syndrome characterized by general inflammation, weight loss and muscle wasting, partly mediated by ubiquitin ligases such as atrogin-1, encoded by *Fbxo32*. Cancers induced by high-risk human papillomavirus (HPV) include anogenital cancers and some head-and-neck cancers and are often associated with cachexia. The aim of this study was to assess the presence of cancer cachexia in HPV16-transgenic mice with or without exposure to the chemical carcinogen 7,12-dimethylbenz(a)anthracene (DMBA). Male mice expressing the HPV16 early region under the control of the cytokeratin 14 gene promoter (K14-HPV16; HPV^+^) and matched wild-type mice (HPV^−^) received DMBA (or vehicle) topically over 17 weeks of the experiment. Food intake and body weight were assessed weekly. The gastrocnemius weights and *Fbxo32* expression levels were quantified at sacrifice time. HPV-16-associated lesions in different anatomic regions were classified histologically. Although unexposed HPV^+^ mice showed higher food intake than wild-type matched group (*p* < 0.01), they presented lower body weights (*p* < 0.05). This body weight trend was more pronounced when comparing DMBA-exposed groups (*p* < 0.01). The same pattern was observed in the gastrocnemius weights (between the unexposed groups: *p* < 0.05; between the exposed groups: *p* < 0.001). Importantly, DMBA reduced body and gastrocnemius weights (*p* < 0.01) when comparing the HPV^+^ groups. Moreover, the *Fbxo32* gene was overexpressed in DMBA-exposed HPV^+^ compared to control mice (*p* < 0.05). These results show that K14-HPV16 mice closely reproduce the anatomic and molecular changes associated with cancer cachexia and may be a good model for preclinical studies concerning the pathogenesis of this syndrome.

## 1. Introduction

Between 50 and 80% of sexually active people worldwide have already had contact with human papillomavirus (HPV), a common sexually transmitted infection [1,2]. The causative role of high-risk (HR) HPV in cervical cancer has been recognized since the 1970s and is considered one of the major infectious causes of cancer, not only in women but also in men [3,4]. Lesions caused by HR HPV (e.g., HPV16 and HPV18) may persist when it is not cleared by the host’s immune response [5]. Lesion persistence increases the risk of developing cervical cancer but also other anogenital cancers (vulvar, vaginal, penile and anal cancers) and a subset of head-and-neck cancers, especially squamous cell carcinomas located in the oropharynx [3,6,7].

Up to 80% of cancer patients can suffer from a multifactorial syndrome named cachexia [8]. Cancer patients with cachexia present weight loss and a loss of skeletal muscle mass with or without a loss of fat mass that cannot be fully reversed by conventional nutritional support [9,10]. One of the pathways that leads to skeletal muscle loss in cancer cachexia is the ubiquitin-mediated proteasome degradation pathway [8,10,11,12]. During cancer cachexia, this pathway is specifically upregulated in skeletal muscle cells, through the expression of ubiquitin ligases such the Atrogin-1, encoded by the *Fbxo32* gene [8,10,11,13]. These ligases have increased expression due to the activation of forkhead box O (FOXO) transcription factors by cytokine-activated NF-κB signaling [8,10,11,13]. The oncoproteins of high-risk HPV can activate NF-κB signaling in multiple ways, contributing to tumorigenesis and cachexia [14,15]. Cancer cachexia is a marker of unfavorable prognosis that is associated with high mortality rates and limits therapeutic options because some types of chemotherapy may further aggravate this syndrome [8,11,16].

K14-HPV16 transgenic mice (HPV^+^) have inserted in their genome the early genomic region of HPV16 [17]. In these animals, the expression of the HPV16 early region is under the control of the cytokeratin-14 (K14) gene promoter, therefore targeting the basal cells of keratinized epithelia [18]. These transgenic mice develop all the typical stages of HPV-induced multistep carcinogenesis in keratinized epithelia, as observed in human patients [19,20]. In fact, these mice present HPV16-induced lesions in different locations besides the uterine cervix, and advanced lesions were recently identified in characteristic head-and-neck sites [21,22,23,24]. Interestingly, the presence of a wasting syndrome in HPV^+^ female mice has also been suggested by our group [14,25].

7,12-dimethylbenz(a)anthrancene (DMBA) is a polycyclic aromatic hydrocarbon found in high concentrations in the tar fraction of cigarette smoke, as well as in car exhaust and furnace gases [26]. DMBA is a carcinogen capable of synergizing with HPV’s oncoproteins to induce carcinomas [27,28], but its association with cancer cachexia remains poorly defined [29,30].

In the present study, we aimed to assess the presence of cancer cachexia in male K14-HPV16 transgenic mice, determining the potential of this animal model for translational studies on cachexia associated with HPV-related cancers. DMBA was employed as a possible cancer promoter and enhancer of this syndrome in mice.

## 2. Results

### 2.1. General Findings

HPV^−^ mice exhibited a normal phenotype without lesions, while the HPV^+^ mice demonstrated several types of macroscopic cutaneous lesions that characterize this animal model, such as auricular erythema, diffuse hyperkeratosis and cephalic alopecia.

### 2.2. Mice Genotyping

**Figure 1:**
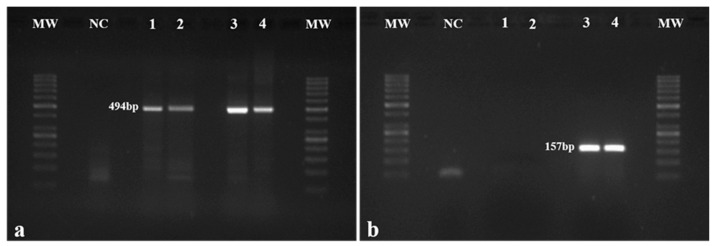
Mouse genotyping. (**a**) *β-globin* gene in mice was amplified by PCR and used as control. *β-globin* amplification (494 bp fragment) was observed in all mice; (**b**) The presence of the HPV16 early region was assessed by the amplification of the HPV16 *E7* gene (157 bp fragment). As expected, amplification was not observed in wild-type mice (Groups 1 and 2 represented by Samples 1 and 2, respectively) but only in HPV^+^ animals (Groups 3 and 4 represented by Samples 3 and 4, respectively). MW: molecular weight marker (GeneRuler 50 bp, Thermo Scientific, Waltham, MA, USA). NC: negative control.

The presence of the HPV16 construct was confirmed through the amplification of the *E7* genomic region (157 bp amplicon) in each mouse (Figure 1b), while the *β-globin* gene (494 bp amplicon) was used as control (Figure 1a). The presence of the HPV16 *E7* was confirmed in all the mice from Groups 3 and 4 (Figure 1b).

### 2.3. Histological Analysis

**Figure 2:**
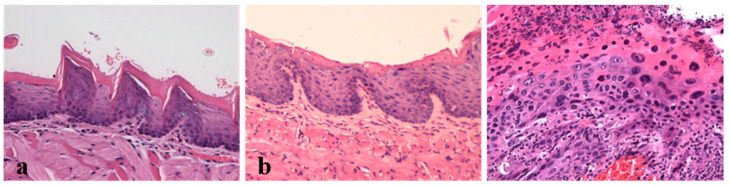
Lesions induced by the HPV16 early genes were analyzed histologically (H&E, 200×). (**a**) Dorsal tongue. Normal oral mucosa (HPV^−^ mouse, Group 1); (**b**) Dorsal tongue. Hyperplastic changes in the oral mucosa (HPV^+^ mouse, Group 3). Note the increased cellularity of the basal layers and normal epithelial differentiation. (**c**) Dorsal tongue. Squamous cell carcinoma (HPV^+^ mouse, Group 3). Note the intense cell pleomorphism and disrupted epithelial architecture.

The evaluation of the chest skin, ear, tongue and penile tissue samples of the wild-type mice (Groups 1 and 2) showed normal histology (Figure 2). By contrast, the tissues of HPV^+^ mice showed a variety of histological lesions, ranging from early hyperplastic to invasive squamous cell carcinomas (Figure 2 and Table 1). While all the sites exhibited intraepithelial hyperplastic and dysplastic changes, invasive squamous cell carcinomas were restricted to the tongue and the ear skin. Hyperplastic lesions of the epidermis and keratinized mucosae showed increased numbers of cell layers and hyperkeratosis without cell atypia. In addition to these features, dysplastic lesions did show cell atypia, including suprabasal mitotic figures, anisokaryosis and disrupted epithelial architecture. Squamous cell carcinomas were well differentiated, with islands of neoplastic keratinocytes invading the underlying stroma, often centered on keratin pearls. DMBA increased the incidence of more severe lesions in the chest skin (*p* = 0.003), in the ear skin (*p* = 0.025) and in the penile mucosa (*p* = 0.006). However, for tongue tissue, a significant difference was not observed (*p* = 0.126).

### 2.4. Body Weight during the Experiment

**Figure 3:**
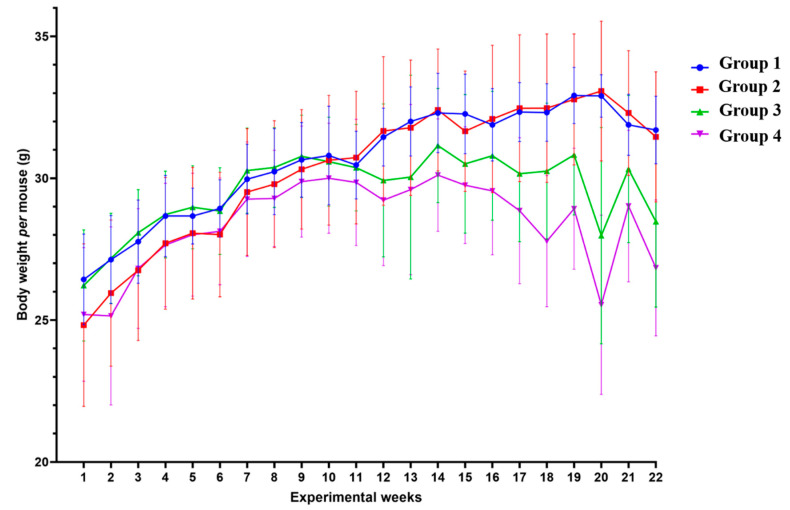
Body weight mean per mouse by group in each week of the experiment. During the 22 weeks of the experiment, transgenic mice (Groups 3 and 4) had lower body weights than matched wild-type mice—Group 3 versus Group 1: *p* < 0.05; Group 4 versus Group 2: *p* < 0.01. Among the HPV^+^ mice, 7,12-dimethylbenz(a)anthracene (DMBA)-exposed animals showed lower body weights—Group 4 versus Group 3: *p* < 0.01. Group 1: HPV^−^ without DMBA application (*n* = 10); Group 2: HPV^−^ with DMBA application (*n* = 10); Group 3: HPV^+^ without DMBA application (*n* = 10); Group 4: HPV^+^ with DMBA application (*n* = 10).

In order to determine if the lesions induced by HPV oncogenes and DMBA induced cachexia in this model, we assessed the body weight of each mouse every week, from the initiation of DMBA application until the mice were sacrificed (Figure 3). During the 22 weeks, HPV^−^ mice without DMBA application (Group 1) showed a median body weight of 31.10 (IQR = 3.38) g/mouse; HPV^−^ mice with DMBA (Group 2) weighed 31.05 (IQR = 4.25) g/mouse; HPV^+^ mice without DMBA application (Group 3) weighed 30.25 (IQR = 1.88) g/mouse, and HPV^+^ mice with DMBA application (Group 4) weighed 28.95 (IQR = 2.22) g/mouse.

From the 11th experimental week (19–21 weeks-old) onwards, we observed that differences between the groups became more noticeable (Figure 3), with the HPV^+^ groups registering lower weights than their HPV^−^ counterparts. Overall, during the 22 weeks of the experiment, the body weights of the HPV^+^ mice were significantly lower than those of matched controls (Groups 1 versus 3: *p* = 0.025; Groups 2 versus 4: *p* = 0.003). Interestingly, there was a pronounced split between the HPV^+^ groups with or without DMBA (Groups 3 and 4), while there were no noticeable differences between the HPV^−^ groups (Groups 1 and 2). Considering the whole experimental period, HPV^+^ mice with DMBA application had a significantly lower body weight than HPV^+^ mice without DMBA (Groups 3 and 4: *p* = 0.003). No statistically significant difference was observed between both HPV^−^ groups (Groups 1 and 2: *p* = 0.860).

**Figure 4:**
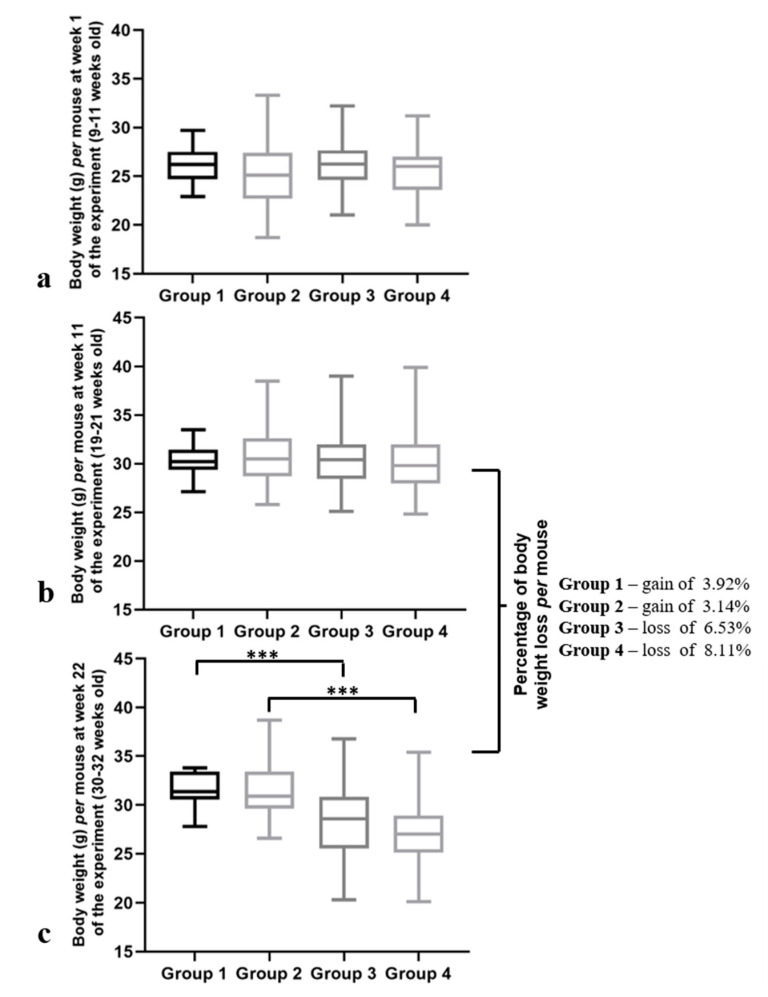
Body weights per mouse at different time points of the experiment. (**a**) Body weight per mouse at Week 1 (9–11 weeks old). Mice of all the groups presented similar body weights at this time point (*p* = 0.100). (**b**) Body weight per mouse at Week 11 (19–21 weeks old). The body weights of all the groups remained similar (*p* = 0.784). (**c**) Body weight per mouse at Week 22 of the experiment (30–32 weeks old). A significant decrease in the body weights of the transgenic mice was observed (*** *p* < 0.001). Comparing the body weights at Weeks 11 and 22, there was a weight gain in the HPV^−^ mice and a weight loss in the HPV^+^ mice (6.5% in Group 3 and 8.1% in Group 4). Group 1: HPV^−^ without DMBA application (*n* = 10); Group 2: HPV^−^ with DMBA application (*n* = 10); Group 3: HPV^+^ without DMBA application (*n* = 10); Group 4: HPV^+^ with DMBA application.

Next, we performed a more detailed analysis of the animals’ bodyweights at various time points. At the first experimental week, (when the mice were 9–11 weeks old) the body weights were similar for all the groups (*p* = 0.100) (Figure 4a). This similarity was preserved until the 11th week of the experiment (19–21 weeks old), where all the groups showed an identical body weight per mouse (*p* = 0.784) (Figure 4b). However, at the 22nd week, differences in the body weights per mouse between the groups were verified (*p* < 0.001) (Figure 4c). Additionally, a significant decrease in the body weights in both HPV^+^ groups were observed when they were compared with matched controls (*p* < 0.001) (Figure 4c). When we compared the body weights at Week 11 of the experiment (when the mice were 19–21 weeks old) to the ones at Week 22 (when the mice were 30–32 weeks old), we observed important differences between the groups: while the HPV^−^ mice (Groups 1 and 2) not only maintained but also gained body weight, the HPV^+^ mice lost 6.5% and 8.1% (Group 3 and group 4, respectively) of their body weight in this period (Figure 4b,c).

### 2.5. Body and Gastrocnemius Weights at the Time of the Sacrifice

**Figure 5:**
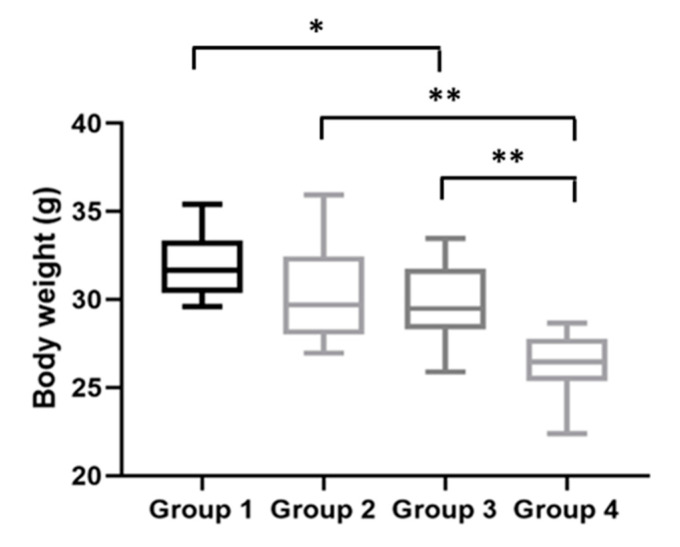
Body weights at the time of sacrifice. HPV^+^ mice had lower body weights than HPV^−^ mice: Group 3 had a statistically significantly lower body weight than Group 1 (* *p* < 0.05); Group 4 had a statistically significantly lower body weight than Group 2 (** *p* < 0.01). Between the HPV^+^ mice, mice with DMBA (Group 4) had a lower body weight than mice without DMBA (Group 3) (** *p* < 0.01). Group 1: HPV^−^ without DMBA application (*n* = 10); Group 2: HPV^−^ with DMBA application (*n* = 10); Group 3: HPV^+^ without DMBA application (*n* = 10); Group 4: HPV^+^ with DMBA application (*n* = 10).

Finally, body weights were assessed immediately before the mice were humanly euthanized. At this final time point, the HPV^−^ mice without DMBA (Group 1) weighed 31.66 (IQR = 2.97) g/mouse; the HPV^−^ mice with DMBA (Group 2) weighed 29.69 (IQR = 4.45) g/mouse; the HPV^+^ mice without DMBA (Group 3) weighed 29.48 (IQR = 3.44) g/mouse; and the HPV^+^ mice with DMBA (Group 4) weighed 26.44 (IQR = 2.39) g/mouse. At the time of sacrifice, the HPV^+^ mice maintained significantly lower body weights than matched HPV^−^ controls (Figure 5). The HPV^−^ mice without DMBA (Group 1) had significantly higher body weights than the HPV^+^ mice without DMBA (Group 3) (*p* = 0.041). There was also a statistically significant difference between the HPV^−^ and HPV^+^ mice with DMBA application (*p* = 0.002) and between both HPV^+^ groups (with and without DMBA) (*p* = 0.005).

**Figure 6:**
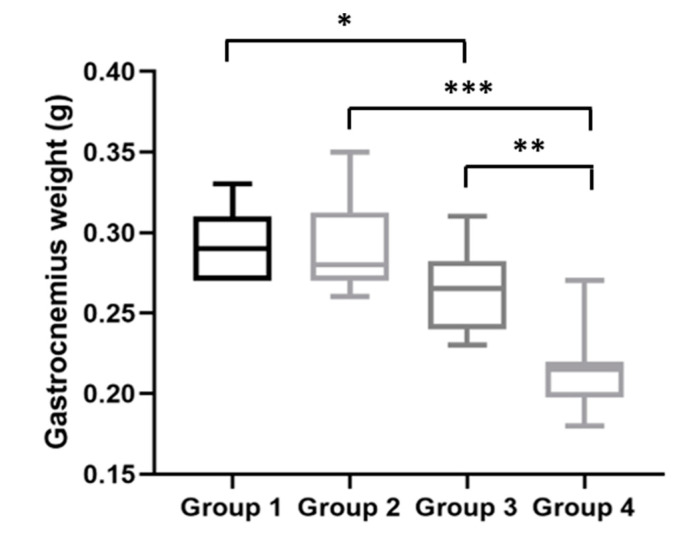
Gastrocnemius muscle weights at the time of sacrifice. HPV^+^ mice had inferior gastrocnemius weights than matched HPV^−^ mice—Group 3 versus Group 1: * *p* < 0.05; Group 4 versus Group 2: *** *p* < 0.001. Additionally, Group 4 had a lower gastrocnemius weight than Group 3 (** *p* < 0.01). Group 1: HPV^−^ without DMBA application (*n* = 10); Group 2: HPV^−^ with DMBA application (*n* = 10); Group 3: HPV^+^ without DMBA application (*n* = 10); Group 4: HPV^+^ with DMBA application (*n* = 10).

After the mice were sacrificed, their gastrocnemius muscles weights were assessed. HPV^−^ mice without DMBA (Group 1) had a median gastrocnemius muscle weight of 290.0 (IQR = 40.0) mg; HPV^−^ mice with DMBA (Group 2), 280.0 (IQR = 40.0) mg; HPV^+^ mice without DMBA (Group 3), 265.0 (IQR = 40.0) mg; and HPV^+^ mice with DMBA (Group 4), 215.0 (IQR = 20.0) mg. Interestingly, at this time point, the gastrocnemius weights were in agreement with the body weights for each group. The HPV^+^ mice (without and with DMBA) had significantly lower muscle weights than matched HPV^−^ mice (Groups 1 versus 3: *p* = 0.025; Groups 2 versus 4: *p* < 0.001) (Figure 6). Additionally, we observed a statistically significant difference (*p* = 0.001) between both HPV^+^ groups, where HPV^+^ mice with DMBA (Group 4) had a lower gastrocnemius weight.

**Figure 7:**
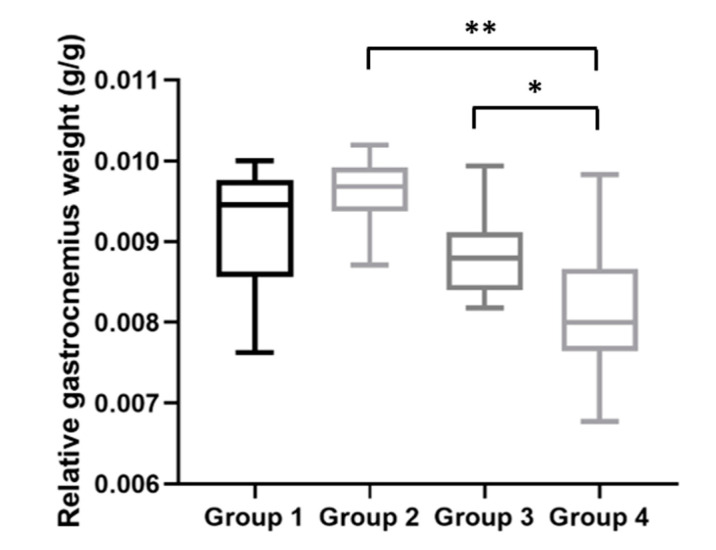
Relative gastrocnemius weights at the time of sacrifice. HPV^+^ mice with DMBA (Group 4) presented a lower relative gastrocnemius weight than matched HPV^−^ mice (Group 2) (** *p* < 0.01). There also was a statistically significant difference between the HPV^+^ groups (Group 4 versus Group 3: * *p* < 0.05). Group 1: HPV^−^ without DMBA application (*n* = 10); Group 2: HPV^−^ with DMBA application (*n* = 10); Group 3: HPV^+^ without DMBA application (*n* = 10); Group 4: HPV^+^ with DMBA application (*n* = 10).

In addition, with the body and gastrocnemius weight data obtained at the time of the sacrifice, we calculated the relative gastrocnemius weight for each mouse (Figure 7). The average relative gastrocnemius weight for HPV^−^ mice without DMBA (Group 1) was 9.46 (IQR = 1.20) mg; for HPV^−^ mice with DMBA (Group 2), 9.68 (IQR = 0.55) mg; for HPV^+^ mice without DMBA (Group 3), 8.80 (IQR = 0.71) mg; and for HPV^+^ mice with DMBA (Group 4), 8.00 (IQR = 1.01) mg. The relative gastrocnemius weights results were in agreement with the absolute weights (Figure 6), and we observed statistically significant differences between Groups 2 and 4 (*p* = 0.002) and between both HPV^+^ groups (*p* = 0.028).

### 2.6. Fbxo32 Expression in Mice Gastrocnemius

**Figure 8:**
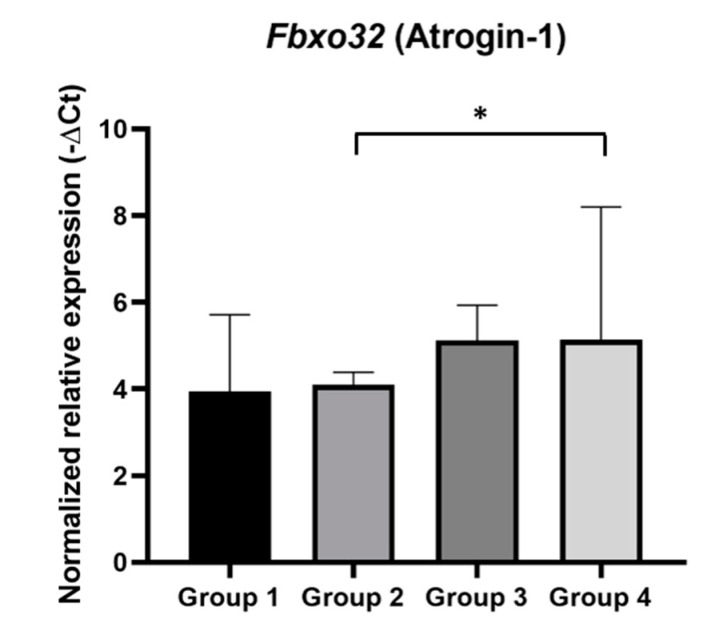
Normalized relative expression of *Fbx32* mRNA in mice gastrocnemius samples. The *Fbxo32* mRNA expression was normalized using the combined expression of the control genes, *Hprt* and *Tbp*. *Fbxo32* expression was significantly increased in HPV^+^ mice with DMBA (Group 4) (* *p* < 0.05). Group 1: HPV^−^ without DMBA application (*n* = 10); Group 2: HPV^−^ with DMBA application (*n* = 10); Group 3: HPV^+^ without DMBA application (*n* = 10); Group 4: HPV^+^ with DMBA application (*n* = 10).

We evaluated *Fbxo32* gene expression in gastrocnemius muscle samples (Figure 8), since it has been considered a marker of muscle atrophy and its expression is associated with the proteolytic machinery in muscle wasting. This analysis showed that *Fbxo32* mRNA expression was significantly increased in HPV^+^ mice with DMBA (Group 4) when compared with that in matched controls (Group 2) (*p* = 0.03). Using the Livak Method, the HPV^+^ mice with DMBA (Group 4) were found to express *Fbxo32* at a 3.62-fold higher level than the HPV^−^ mice with DMBA (Group 2). In HPV^+^ mice without DMBA (Group 3), the average expression of *Fbxo32* also seemed to slightly increase when compared with that in HPV^−^ mice without DMBA (Group 1), but this difference did not reach statistical significance (*p* = 0.55). Additionally, there were no significant differences between the HPV^−^ groups (Groups 1 and 2: *p* = 0.78) or between the HPV^+^ groups (Groups 3 and 4: *p* = 0.68), although Group 4 showed a higher average expression of *Fbxo32*.

### 2.7. Food Intake during the Experiment

**Figure 9:**
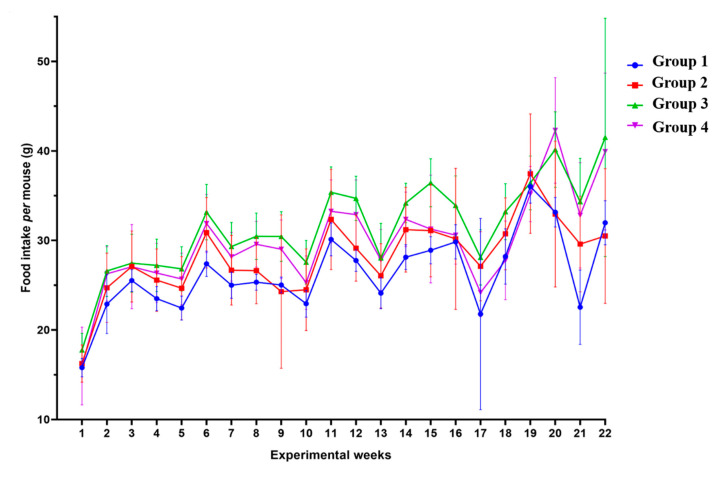
Weekly food intake per mouse by group. During the 22 weeks of the experiment (DMBA application from Week 1 until Week 17 of the experiment), it seems that the HPV^+^ mice (Groups 3 and 4) had a higher consumption of food (per mouse) than the HPV^−^ ones (Groups 1 and 2). However, there only exists a statistically significant difference between Groups 1 and 3 (*p* < 0.01). Group 1: HPV^−^ without DMBA application (*n* = 10); Group 2: HPV^−^ with DMBA application (*n* = 10); Group 3: HPV^+^ without DMBA application (*n* = 10); Group 4: HPV^+^ with DMBA application (*n* = 10).

To confirm whether the loss of body weight and skeletal muscle mass in the HPV^+^ mice could be attributed to lower food ingestion, we analyzed the weekly food intake for each group (Figure 9). We observed that the HPV^−^ mice without DMBA application (Group 1) ingested 26.29 (SD = 4.46) g/mouse/week, during the 22 experimental weeks; the HPV^−^ mice exposed to DMBA (Group 2) ingested 28.16 (SD = 4.27) g/mouse/week; the HPV^+^ mice without DMBA application (Group 3) showed the highest food intake at 31.51 (SD = 5.30) g/mouse/week; and in the HPV^+^ mice with DMBA application (Group 4), the food intake was 29.80 (SD = 5.50) g/mouse/week. The HPV^+^ mice showed a higher food consumption than matched HPV^−^ mice. However, the only statistically significant difference (*p* = 0.001) was found between HPV^−^ without DMBA application (Group 1) and HPV^+^ without DMBA (Group 3) mice.

## 3. Discussion

Despite multiple attempts to understand its pathophysiology and develop therapeutic strategies, cancer cachexia remains a common and challenging syndrome [31,32]. While in cases of anorexia or starvation, the loss of skeletal muscle mass may be restored with adequate therapeutic intervention, the sarcopenia that characterizes cancer cachexia cannot be fully reversed by conventional nutritional support [9,10]. The most consensual diagnostic criterion for cachexia is a weight loss of over 5% over 6 months or over 2% in individuals with a body-mass index (BMI) below 20 or with sarcopenia [9]. Importantly, cancer cachexia contributes to at least 20% of cancer-associated deaths [8,31]. Weight loss is a prognostic factor associated with poor responses to chemoradiotherapy and reduced survival among cancer patients [32].

**Figure 10:**
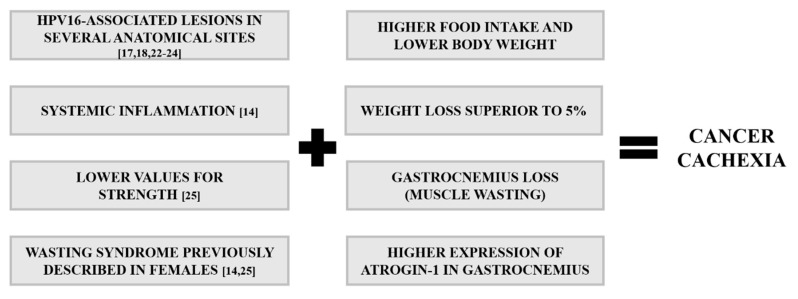
Key features of cancer cachexia in K14-HPV16 mice. Lesions associated with HPV16, ranging from hyperplasia to carcinoma, occur in typical anatomical sites. Systemic inflammation, low values of muscle strength and muscle wasting occur in K14-HPV16 transgenic mice. These events help to validate this model for studying cancer cachexia.

The lack of in-depth knowledge about cachexia’s pathophysiology remains a limiting factor for the diagnosis and treatment of this syndrome [8,33,34,35]. Animal models play an essential role in translational research and preclinical tests for novel therapies [14,25,36], and a number of them are available for studying cancer cachexia, predominantly tumor xenograft and syngeneic mouse models [37,38]. However, these types of models present significant limitations including their short time courses (2–25 days), often-extreme tumor burdens and excessive inflammatory status phenotypes [39,40]. Additionally, the injection site of tumor cells in the host may cause unwanted variations in the cachectic phenotype [39,40]. In fact, the animal models currently used are characterized by an abrupt and rapid cachexia with deadly wasting syndrome within a short period of time, while cachectic patients suffer from progressive alterations [41]. Therefore, it would be desirable to have a model of progressive tumor development with a more extended time course that could mimic more closely the changes observed in cancer patients [42]. In order to achieve this, genetically engineered mouse models could be a good approach, such the KPP mouse, which develops progressive cachexia associated with advancing pancreatic pathology [43]. The K14-HPV16 mice used in this study could also provide such a model of progressive development [17,18]. We recently described that these mice develop severe systemic inflammation coupled with reduced grip strength, which are important features of cachexia (Figure 10) [14,25]. The present results help to further characterize this model, describing key molecular and morphological characteristics that help to validate it for the study of cancer cachexia.

A good cancer cachexia model must present well-defined tumor lesions. The histological evaluation showed that the HPV^+^ mice presented histological lesions in anatomical sites typically affected by HPV16, such as the tongue base and the penis, and also on cutaneous sites typically involved in this mouse model. DMBA application was useful for inducing more severe penile lesions and also seems to have promoted carcinogenesis in other anatomical sites, presumably due to the animal’s grooming behavior, which is likely to help in spreading the carcinogen. This is consistent with previously described histological lesions [17,18,21,23,24] and provides a framework for studying cancer cachexia in this model. The fact that DMBA promoted tumor lesions is consistent with the fact that DMBA-exposed transgenic animals showed a more severe cachectic phenotype than matched untreated mice. In fact, HPV^+^ mice with DMBA application lost more weight than HPV^+^ mice without DMBA, in line with the more severe lesions.

We observed that HPV^+^ mice had higher food intakes than wild-type controls, which leads us to conclude that the decreased body weight in the transgenic mice was not due to anorexia or reduced food availability (food was offered ad libitum). In fact, cancer cachexia can be present in patients with or without a decrease in appetite, and nutritional support is insufficient to prevent weight loss [8,11]. In addition, several other animal models present a loss of skeletal muscle, adipose tissue and body weight without reduced food intake [37,39,44,45]. Body weight differences became apparent by the 11th experimental week, which coincides with the onset of the more severe dysplastic lesions in this model, as previously described [19,46]. Both HPV^+^ groups (with and without DMBA) showed weight losses over 5%, which fits the criteria for the clinical diagnosis of cancer cachexia [9] and supports the use of K14-HPV16 for studying this syndrome.

Importantly, skeletal muscle wasting followed the same pattern as total body weight and was associated with *Fbxo32* up-regulation, adding more similarities between this model and the syndrome observed in cancer patients [40,47]. The *Fbxo32* gene encodes the protein atrogin-1, which has already been demonstrated to be an E3 ubiquitin ligase that is upregulated in muscle atrophy and that may be a potential molecular target for treating muscle atrophy induced by cancer cachexia [48,49].

The present results agree with a previous study that described the body weight loss in the *Apc^Min/+^* mice model of cancer [39]. At 3 months of age, *Apc^Min/+^* and wild-type mice in a C57Bl/6 background presented similar body weights and both groups maintained physiological weight gains up to 6 months of age [39]. However, by the end of the experiment, the *Apc^Min/+^* mice presented lower body weights despite higher food intakes [39]. The authors also observed muscle wasting in the *Apc^Min/+^* mice compared to controls [39]. The results from this study also agree with previous data from our group obtained from HPV16-transgenic female mice [14,25]. It would now be interesting to compare the wasting process in males and females using this mouse strain. As the transgenic mouse model used in the mentioned study [39], the K14-HPV16 mice model seems to present the same advantages and to be a good model for the study of cancer cachexia, especially for the study of cachexia in HPV-induced cancers. The weakness of the present study includes the lack of additional molecular markers of cachexia, of a thorough histological examination of muscle tissue and of parameters related to skeletal muscle function. Additional experiments should employ complementary strategies to further validate this model, by studying the expression of other cachexia-associated genes (e.g., *Trim63*, which encodes muscle RING finger 1), determining parameters such as twitch force, tetanic force and eccentric contraction and studying cross-sections of affected muscles histologically.

The differences verified between both HPV^+^ mouse groups were associated with severe lesions induced by DMBA, creating a more prominent cachexia syndrome in the HPV^+^ mice exposed to DMBA. This potent carcinogen was used in numerous animal models [29,50,51], being also associated with weight loss. In one study with hamsters, weight loss was more pronounced in animals with DMBA application [52]. Similar results were obtained in rats [51]. Another study showed that interrupting DMBA application allowed the animals to regain some weight, suggesting an acute toxic effect [53]. Even so, DMBA is not an essential factor for observing this syndrome in the K14-HPV16 mice, as suggested by this work and other works by our group [14,25]. In a recent study, we showed that the topical penile application of DMBA did not induce significant systemic genotoxicity or significant differences in the weights of internal organs [24], presumably because the animal’s grooming behavior was insufficient to promote significant ingestion of the drug.

## 4. Materials and Methods 

### 4.1. Mice

K14-HPV16 transgenic mouse generation on an FVB/n background has been previously reported [18]. These transgenic mice were kindly donated by Dr. Jeffrey Arbeit and Dr. Douglas Hanahan (University of California) through the USA National Cancer Institute Mouse Repository. The animal experiments were approved by the University of Trás-os-Montes and Alto Douro Ethics Committee (10/2013) and the Portuguese General Veterinary Directorate (approval no. 0421/000/000/2014, 24 September 2014). The mice (wild-type and transgenic) were housed and bred according to Portuguese (Decreto-Lei 113, August 7th) and European (EU Directive 2010/63/EU) legislation, under controlled temperature (23 ± 2 °C), light–dark cycle (12h light/12h dark) and relative humidity (50 ± 10%). Food and water were provided ad libitum.

### 4.2. Experimental Design and Sample Collection

**Figure 11:**
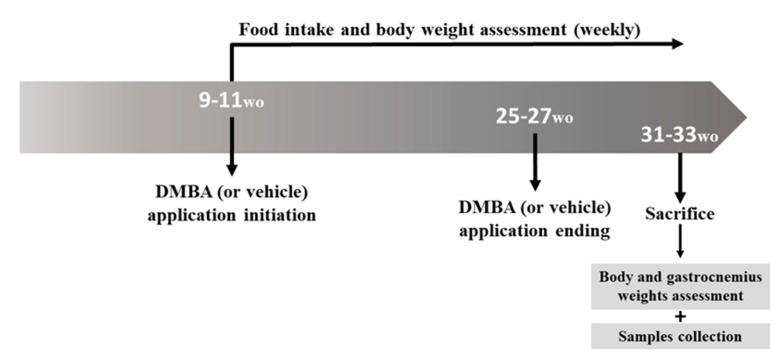
Experimental design timeline. Weekly DMBA application (0.031 mg/animal/week in 4 μL of DMSO) started at 9–11 weeks of age, lasting for 17 weeks. Groups 2 and 4 received DMBA, while Groups 1 and 3 received DMSO only (the vehicle). Animals were sacrificed at 31–33 weeks of age for sample collection. wo: weeks old.

Forty 9–11-week-old male mice were allocated to four groups: Group 1 (*n* = 10, wild-type (HPV^−^) mice, without DMBA application), Group 2 (*n* = 10, HPV^−^ mice, with DMBA application), Group 3 (*n* = 10, HPV^+^ mice, without DMBA application) and Group 4 (*n* = 10, HPV^+^ mice, with DMBA application). The DMBA (D3254, Sigma-Aldrich, Merck kGAa, Darmstadt, Germany) was dissolved in DMSO (CARLO ERBA Reagents S.A.S., Val de Reuil, France) and was topically administered to the penile mucosa once a week (0.031 mg/animal/week in 4 μL of DMSO). DMBA (or vehicle) administration started at 9–11 weeks of age and lasted for 17 consecutive weeks (Figure 11). All the mice were sacrificed at 31–33 weeks of age under ketamine (CLORKETAM 1000, injectable solution, Vétoquinol, Barcarena, Portugal) and xylazine (Rompun^®^ 2%, Bayer Healthcare S.A., Kiel, Alemanha) anesthesia, by intracardiac puncture and exsanguination, as indicated by the Federation for Laboratory Animal Science Associations (FELASA). From the initiation of DMBA application, the food intake and the body weight were weekly registered for all the mice. The total body weights of all the mice were also assessed before they were humanely euthanized, and the gastrocnemius weight was assessed immediately after sacrifice (Figure 11). The gastrocnemius muscle samples were collected in TripleXtractor reagent (Grisp^®^, Porto, Portugal), macerated and kept at −80 °C until further use. Chest skin, ear, penis and tongue samples were also collected for histological analysis.

### 4.3. Mouse Genotyping 

DNA isolation and purification from the samples in TripleXtractor reagent (Grisp^®^) was performed using the GRS Genomic DNA Kit-Broad Range (Grisp^®^). DNA concentration and purity were assessed using the NanoDrop™ Lite spectrophotometer (Thermo Scientific, Waltham, MA, USA). The presence of the HPV16 early region was confirmed by the amplification of the HPV16 *E7* gene, using the mouse *β-globin* gene as a control, through polymerase chain reaction (PCR) with specific primers for both genes (Table S1). The PCR reaction was performed in the GeneAmp^®^ PCR System 9700 thermal cycler (Applied Biosystems^®^, Foster City, CA, USA), in a total volume of 25 μL with 1x PCR Buffer with dye, 0.4 mM dNTPs, 1.5 mM MgCl2, 0.3 μM concentrations of each primer, 0.5 U of Xpert Taq DNA Polymerase (Grisp^®^) and 0.2 μg of genomic DNA. For both genes, the amplification conditions were the following: DNA denaturation at 95 °C for 5 min, followed by 35 cycles at 95 °C for 20 s, 55 °C for 30 s and 72 °C for 1 min, and a final extension at 72 °C for 5 min. For each PCR, the amplified fragments were analyzed by electrophoresis in 1.5% (*w*/*v*) agarose gels stained with GreenSafe Premium (NZYTech, Lisbon, Portugal) and visualized in GelDocXR (Bio-Rad Laboratories, Hercules, CA, USA).

### 4.4. Histological Anlysis 

The chest skin, ear, penis and the tongue samples collected were fixated in 10% neutral buffered formalin. Then, the tissues were dehydrated through graded alcohols and xylene using a Citadel 2000™ Tissue Processor (Thermo Scientific) and embedded in paraffin. Paraffin blocks were cut into 3 μm-thick sections and stained with hematoxylin and eosin (H&E) for evaluation and classification under a bright field microscope. The samples were classified as normal, hyperplastic, dysplastic or squamous cell carcinoma based on changes involving the epidermis or the stratified squamous epithelia of the oral and penile mucosae. Hyperplasia was diagnosed based on an increased number of basal cell layers (over two layers in the epidermis and over 3 layers in the oral and penile mucosae), without cytological atypia. Dysplasia arose in foci from a hyperplastic background but also involved nuclear crowding, the loss of suprabasal differentiation and the presence of cytological atypia. Squamous cell carcinoma was diagnosed based on the invasion of the underlying stroma by groups of neoplastic cells.

### 4.5. Food Intake and Body Weights 

The food in each mouse cage was weighed at the beginning of each week. The food intake per week per animal was calculated using the following formula:

$$\frac{\mathrm{Food}\;week\;n\;\left(g\right)-\mathrm{Food}\;week\;n+1\;\left(g\right)}{\mathrm{Number}\;\mathrm{of}\;\mathrm{animals}\;\mathrm{in}\;\mathrm{the}\;\mathrm{cage}}.$$

Then, the mean food intake per week was calculated for each group, and results are expressed as the mean ± standard deviation.

The percentage of total body weight gained or lost during the experiment per animal was calculated using the following formula:

$$\frac{\mathrm{First}\;\mathrm{week}’s\;\mathrm{weight}\;\left(g\right)-\mathrm{Last}\;\mathrm{week}’s\;\mathrm{weight}\;\left(g\right)}{\mathrm{First}\;\mathrm{week}’s\;\mathrm{weight}\;\left(g\right)}\times 100.$$

### 4.6. Relative Quantification of Fbxo32 mRNA Expression 

Total RNA extraction from gastrocnemius samples was performed using TripleXtractor reagent (Grisp^®^) followed by a chloroform solution (EMSURE^®^, Merck kGAa, Darmstadt, Germany). Total RNA was then purified using a GRS total RNA kit (Grisp^®^), and the concentration and purity were assessed using the NanoDrop™ Lite spectrophotometer (Thermo Scientific). Then, 150 ng of total RNA were reverse transcribed using the High-Capacity cDNA Reverse Transcription Kit (Applied Biosystems^®^), and the thermal conditions were the following: 10 min at 25 °C, 120 min at 37 °C, and 5 min at 8 °C. The cDNA was then used as a template for quantitative real-time PCR (qPCR) using a StepOne qPCR Real-Time PCR device (Applied Biosystems^®^). *B2m*, *Hprt* and *Tbp* were tested as potential endogenous controls, and the combination of *Hprt* and *Tbp* was selected since these two genes showed the lowest standard deviation values. Fast SYBR™ Green Master Mix (Applied Biosystems^®^) was added to the primers for *Hprt*, *Tbp* and *Fbxo32* (Table S2) and to the cDNA sample with the following conditions: 20 s at 95 °C followed by 40 cycles of 3 s at 95 °C and 30 s at the annealing temperature of each pair of primers. The amplification efficiency for each pair of primers was determined using a two-fold serial dilution of cDNA reverse transcribed from Universal Mouse Reference RNA (Invitrogen™, Waltham, MA, USA). Only efficiencies between 90% and 110% were accepted. The same baseline and threshold were set for each plate using the analysis software for qPCR from the Thermo Fisher Connect platform (Thermo Fisher Scientific, Waltham, MA, USA), in order to generate threshold cycle (Ct) values for all the genes in each sample.

### 4.7. Statistical Analysis 

Statistical analysis was performed using IBM SPSS Statistics for Windows (Version 25.0). Any statistical differences in the histological analysis of mouse tissue were evaluated using Fisher’s exact test or the chi-square test. Prior to evaluating any statistical differences between two groups in terms of food intake and total body weight during the 22 weeks of the experiment, the normality of the data was tested (Shapiro–Wilk test). When the data followed a normal distribution, the data were presented as mean and standard deviation (SD) and a Student’s *t* test was performed; otherwise, the data were expressed as median and inter-quartile range (IQR) and a Mann–Whitney U test was used. Moreover, the presence of statistical differences in the body weights among the four groups at specific weeks of the experiment was evaluated using the ANOVA test, since the data were normally distributed. The values of the gastrocnemius and total body weights assessed at the time of the sacrifice were presented as the median and IQR and were evaluated using the Mann–Whitney U test. *Fbxo32* expression was evaluated using the Livak method along with the Mann–Whitney U test. All the graphics were constructed using GraphPad Prism 8 (GraphPad Software). The results were considered statistically significant when the *p* values were <0.05.

## 5. Conclusions

In the present study, we hypothesized that DMBA could enhance the wasting syndrome induced by the HPV16 oncogenes in mice. This hypothesis is supported by our results, showing that DMBA promoted the development of more severe lesions in association with inferior gastrocnemius and body weights. Even so, DMBA is not an essential factor for observing this syndrome in the K14-HPV16 mice. Additionally, there were no statistical differences between the HPV^−^ mice with or without DMBA application, indicating that DMBA per se does not induce cachexia, but acts by enhancing the carcinogenesis initiated by the HPV16 oncogenes.

Therefore, we suggest that K14-HPV16 mice are a good model for studying the pathophysiology of cachexia associated with HPV-induced cancers and a useful tool for the preclinical testing of new therapies.

## Figures and Tables

**Table 1:** Histological classification of mouse samples.

Group	Anatomic Regions	Histological Classification (%)			
		Normal	Hyperplastic Lesions	Dysplastic Lesions	Invasive Squamous Cell Carcinoma
1 (HPV^−^ without DMBA application, *n* = 10)	Chest skin	10/10 (100%)	0/10 (0%)	0/10 (0%)	0/10 (0%)
	Ear skin	10/10 (100%)	0/10 (0%)	0/10 (0%)	0/10 (0%)
	Penile	10/10 (100%)	0/10 (0%)	0/10 (0%)	0/10 (0%)
	Tongue	10/10 (100%)	0/10 (0%)	0/10 (0%)	0/10 (0%)
2 (HPV^−^ with DMBA application, *n* = 10)	Chest skin	10/10 (100%)	0/10 (0%)	0/10 (0%)	0/10 (0%)
	Ear skin	10/10 (100%)	0/10 (0%)	0/10 (0%)	0/10 (0%)
	Penile	10/10 (100%)	0/10 (0%)	0/10 (0%)	0/10 (0%)
	Tongue	10/10 (100%)	0/10 (0%)	0/10 (0%)	0/10 (0%)
3 (HPV^+^ without DMBA application, *n* = 10)	Chest skin	0/10 (0%)	10/10 (100%)	0/10 (0%)	0/10 (0%)
	Ear skin	0/10 (0%)	8/10 (80%)	2/10 (20%)	0/10 (0%)
	Penile	2/10 (20%)	7/10 (70%)	1/10 (10%)	0/10 (0%)
	Tongue	1/10 (10%)	4/10 (40%)	4/10 (40%)	1/10 (10%)
4 (HPV^+^ with DMBA application, *n* = 10)	Chest skin	0/10 (0%)	3/10 (30%)	7/10 (70%)	0/10 (0%)
	Ear skin	0/10 (0%)	2/10 (20%)	7/10 (70%)	1/10 (10%)
	Penile	0/10 (0%)	2/10 (20%)	8/10 (80%)	0/10 (0%)
	Tongue	0/10 (0%)	9/10 (90%)	1/10 (10%)	0/10 (0%)

## Supplementary Materials

The following are available online at https://www.mdpi.com/1422-0067/21/14/5020/s1. Table S1: Primers Sequences for Mice Genotyping. Table S2: Primers Sequences used for qPCR.
