# Supplementary material for: Human Papillomavirus 16-Transgenic Mice as a Model to Study Cancer-Associated Cachexia
Source: Int J Mol Sci. 2020 Jul 16;21(14):5020. doi: 10.3390/ijms21145020 (PMC7404304; doi:10.3390/ijms21145020)
Supplement: Supplementary file 1 [file ijms-21-05020-s001.pdf]

Supplementary Table S1. Primer Sequences for Mice Genotyping.

Primer Target Gene		Sequence (5′- 3′)	Amplicon size
**HPV16 *E7***	FORWARD	GGAGGAGGATGAAATAGATGG	157 bp
	REVERSE	GCCCATTAAGAGGTCTTCCAA	
β-globin	FORWARD	CCAATCTGCTCACACAGGATAGAGAGGGCAGG	494 bp
	REVERSE	CCTTGAGGCTGTCCAAGTGATTCAGGCCATCG	

Supplementary Table S2. Primers Sequences used for qPCR.

Gene Name	Gene Product	Primers Sequences (5′- 3′)	
Hprt	Hypoxanthine-guanine phosphoribosyltransferase	FORWARD	TGAAGAGCTACTGTAATGATCAGTCAAC
		REVERSE	AGCAAGCTTGCAACCTTAACCA
Tbp	TATA binding protein	FORWARD	CAAACCCAGAATTGTTCTCCTT
		REVERSE	ATGTGGTCTTCCTGAATCCCT
Fbxo32	Atrogin-1	FORWARD	GGGTGTATCGGATGGAGACC
		REVERSE	GTTGTAAGCACACAGGCAGG
